# Endocrine disruption in teleosts and amphibians is mediated by anthropogenic and natural environmental factors: implications for risk assessment

**DOI:** 10.1098/rstb.2022.0505

**Published:** 2024-03-25

**Authors:** Werner Kloas, Matthias Stöck, Ilka Lutz, Andrea Ziková-Kloas

**Affiliations:** ^1^ Department of Fish Biology, Fisheries and Aquaculture, Leibniz Institute of Freshwater Ecology and Inland Fisheries, Müggelseedamm 310, 12587 Berlin, Germany; ^2^ Department of Endocrinology, Institute of Biology and Albrecht Daniel Thaer Institute, Faculty of Life Sciences, Humboldt University, Unter den Linden 6, 10117 Berlin, Germany; ^3^ Ecotoxicological Laboratory, German Environment Agency, Schichauweg 58, 12307 Berlin, Germany

**Keywords:** endocrine disruptors (EDs), amphibians, fishes, environmental pollution, parasites, artificial light at night (ALAN)

## Abstract

Environmental variation in the Anthropocene involves several factors that interfere with endocrine systems of wildlife and humans, presenting a planetary boundary of still unknown dimensions. Here, we focus on chemical compounds and other impacts of anthropogenic and natural origins that are adversely affecting reproduction and development. The main sink of these endocrine disruptors (EDs) is surface waters, where they mostly endanger aquatic vertebrates, like teleost fish and amphibians. For regulatory purposes, EDs are categorized into EATS modalities (oestrogenic, androgenic, thyroidal, steroidogenesis), only addressing endocrine systems being assessable by validated tests. However, there is evidence that non-EATS modalities—and even natural sources, such as decomposition products of plants or parasitic infections—can affect vertebrate endocrine systems. Recently, the disturbance of natural circadian light rhythms by artificial light at night (ALAN) has been identified as another ED. Reviewing the knowledge about EDs affecting teleosts and amphibians leads to implications for risk assessment. The generally accepted WHO-definition for EDs, which focuses exclusively on ‘exogenous substances' and neglects parasitic infections or ALAN, seems to require some adaptation. Natural EDs have been involved in coevolutionary processes for ages without resulting in a general loss of biodiversity. Therefore, to address the ‘One Health’-principle, future research and regulatory efforts should focus on minimizing anthropogenic factors for endocrine disruption.

This article is part of the theme issue ‘Endocrine responses to environmental variation: conceptual approaches and recent developments’.

## 1. Introduction

Chemical communication is the basic concept for all organisms to exchange information endogenously, between individuals, and the environment [1,2]. In animals, three major abilities for such communication have evolved, namely the immune, nervous and endocrine systems. In a complex interplay, these systems respond to environmental variation and especially the endocrine system triggers—via short- and midterm actions of hormones and their corresponding receptors—important physiological processes, e.g. reproduction, development, metamorphosis, colour change, osmomineral regulation, behaviour or metabolism. The endocrine systems of various animal groups have evolved to cope and mediate successfully in acclimatizing, adaptating and surviving environmental variation within their natural ranges over the ages. After the strong climatic oscillations of the Pleistocene [3], relatively stable and convenient conditions during the following Holocene [4] fostered the successful development of human civilization, and with the industrial revolution, this finally led to a new era—the Anthropocene [5]—which was radically driven by human activities. These impacts provoked even more rapid environmental alterations, e.g. climate change. Severe warnings were issued that mankind is exceeding the limits of the planet’s ability to compensate for our actions, with the likelihood that many geographical regions—or even the whole Earth—will be detrimentally effected [6]. Although we have started to develop a worldwide system of justice for the human population, along with conservation efforts towards other species and the planet, it seems obvious that the red lines of several planetary boundaries have already been crossed and that drastic efforts are required to cope with the major and rapid changes that we have already induced [7].

One of these planetary boundaries comprises chemical pollution but due to the complex mixtures of thousands of compounds released and still leaking into the environment, it is not really quantifiable where the red line of that boundary might be [6,7]. Thus, a sound prediction of how chemical pollution adversely interferes with living organisms is hard to make. This requires new concepts for such novel entities to cope with in a sustainable Anthropocene [8]. In this respect, it might be beneficial to assist and apply the ‘One Health’ approach, aiming to sustainably balance and optimize the health of people, animals and ecosystems [9].

The best example demonstrating the judgment complexity in performing a sound risk assessment of the present chemical pollution concerns the so-called endocrine disruptors (EDs). An ED is defined as ‘an exogenous substance or mixture that alters function(s) of the endocrine system and consequently causes adverse health effects in an intact organism, or its progeny, or (sub-)populations’ and a ‘potential endocrine disruptor is an exogenous substance or mixture that possesses properties that might be expected to lead to endocrine disruption in an intact organism, or its progeny, or (sub-)populations' [10]. These definitions reveal that ED research focuses mainly on endocrine interference caused by chemical compounds that are released into the environment and threaten wildlife or cause damage to humans, e.g. via water, diets or personal care products. The majority of investigations concerning ED effects have been performed using mammalian models or epidemiological studies to assess potential threats for humans and other vertebrate models to determine impacts on wildlife. However, due to the high degree of conservation of endocrine systems across all classes of vertebrates, ED research has focused on vertebrates, using representative models of wildlife as sentinels for human and environmental health. Although invertebrates comprise by far the majority of animal species, and despite some similarities to vertebrate systems might exist in various invertebrate classes, highly differentiated endocrine systems have not evolved or remain undetected. However, in principle, it is of great concern that endocrine disruption may cause adverse impacts in invertebrates, but this still appears somewhat underestimated [11,12].

Although the WHO definition of EDs is now generally accepted and probably covers the majority of sources for EDs, it can be stated that disruption of endocrine systems is not only mediated by exogenous substances or their mixtures. In addition, further environmental factors beyond the framework of that definition might contribute to act as EDs.

Therefore, the aim of this review is to provide a comprehensive overview of endocrine disruption in teleost fishes and amphibians, since surface waters present the main sink for EDs, and to present sensitive vertebrate sentinels in context with the ‘One Health’ approach. The focus is to provide evidence that, besides substances of anthropogenic or natural origin, known to act as EDs, other environmental factors, such as temperature, light and diseases (e.g. parasites), are also impacting endocrine systems, which suggests that partial modifications for the existing WHO definition for EDs appear recommended. We will link our short overview to existing tests and relevant guidelines and deduce implications and future research requirements suggested by shortcomings of current approaches.

## 2. Endocrine disruption in teleosts and amphibians

The endocrine systems of fishes and amphibians are remarkably similar to those of mammals, including humans; however, some differences in endocrine regulation exist [13]. Fishes are the most ‘primitive’ vertebrates, and are divided into three classes: *Cyclostomes* (jawless), *Chondrichthyes* (cartilaginous) and *Osteichthyes* (bony). In surface waters, teleosts—the largest infraclass of bony fishes—prevail and thus present reasonable sentinels and model organisms to assess EDs. The vertebrate transition to partial terrestrial life style is only represented by the vertebrate class *Amphibia*, including the three orders *Anura* (frogs), *Urodela* (salamanders) and *Gymnophiona* (caecilians). Although information about endocrine functions is available for all three orders, ED research has mostly been performed in frogs as classical models for endocrinology and developmental biology.

The original hypothesis that EDs might play important roles to adversely alter vertebrate reproduction, especially via oestrogenic impacts on male individuals, has been developed nearly in parallel for humans [14], wildlife including fish [15] and amphibians [16]. That hypothesis fostered not only toxicological but also related environmental research, generating valuable data to reveal that not only EDs with oestrogenic modes of action (MOA) might exist. This initiated implications for environmental risk assessments by the European Union in the framework of REACH (Registration, Evaluation and Authorisation of CHemicals) [17,18]. The first reviews compiled early findings on ED for fishes [19] and amphibians [20], with extensions during following years by further MOA, affecting the reproductive endocrine systems [21], and metamorphosis as triggered by the thyroid system [22]. Rising awareness concerning EDs and their potential to adversely affect vertebrate endocrine systems led to a broader development of test guidelines within the framework of the OECD (Organization of Economic Cooperation and Development) that proposed a set of testing methods to assess EATS (oestrogenic, androgenic, thyroidal, steroidogenic) modalities *in vivo* and *in vitro*.

In addition, a conceptual framework of adverse outcome pathways (AOP) has been introduced, even exemplifying oestrogenic modalities, to facilitate ecotoxicological research and risk assessment [23]. Furthermore, ECHA (European Chemicals Agency) and EFSA (European Food Safety Authority) developed a guidance document [24] explaining how to assess EATS modalities for regulatory purposes. From an endocrinological point of view, it is obvious that EDs in vertebrates are not restricted only to EATS; they also impact reproduction, metamorphosis and development, but might affect further endocrine systems as well [24–26] by non-EATS modalities. In addition, the OECD compiled the recent knowledge about endocrine disruption in freshwater [27], providing hints for a conceptual framework to address this issue for regulatory purposes.

Therefore, we will review our recent knowledge on ED in teleosts and amphibians by distinguishing EATS and non-EATS modalities, as well as by providing evidence that besides exogenous substances, further environmental factors that are not covered by the recent WHO definition for ED might also cause endocrine disruption.

## 3. EATS (oestrogenic, androgenic, thyroidal, steroidogenic) modalities

Starting out from the ‘oestrogen hypothesis' [14,15,28] as a phenomenon and medical syndrome of endocrine disruption, it soon became evident that beside oestrogenic MOA further modalities exist including activation/inhibition of oestrogen, androgen and thyroid receptor signalling, as well as bioavailability of steroids or thyroid hormones, nicely summarized as EATS [29,30].

To revise the fundamentals of how anthropogenic and natural substances might interfere via EATS in aquatic vertebrates, we first briefly review some of the recent findings about reproduction, covering oestrogenic, androgenic and steroidogenic MOA as well as metamorphosis/development triggered by thyroidal MOA.

### (a) Overview of the reproductive biology of teleosts and amphibians in context to EATS

Reproductive biology of most vertebrates covers two major phases: sexual differentiation of developing individuals and maintenance of reproductive functions in adults. In gonochoristic vertebrates, genetic and cellular processes underlying sexual differentiation exhibit great diversity and sex determination ranges from environmental to simple or complex genetic systems [31]. ED risk assessment of aquatic vertebrates is determined for the teleost models zebrafish (*Danio rerio*), fathead minnow (*Pimephales promelas*), the medaka fish (*Oryzias latipes*) and the threespined stickleback (*Gasterosteus aculeatus*) as well as for the anuran models *Xenopus laevis* and tropical clawed frog (*Xenopus tropicalis*). Thus, this survey about the reproductive biology also remains restricted to teleosts and frogs.

Teleosts cover the largest diversity of reproductive modes [32], including all-female sperm-dependent parthenogenetic (=‘gynogenetic’) [33], sequential (protandrous, protogynous or serial) hermaphroditism [34], and in some cases even involving socially controlled sex change [35] or simultaneous hermaphroditism [36]. Sexual development of teleosts is characterized by a high degree of plasticity [37], and sex reversal can be easily induced by hormonal and sometimes even by environmental triggers [38], fostering such teleosts to serve as model species for ED [39]. Beside environmental sex determination and a high plasticity to environmental triggers (e.g. ED), genetic (= genotypic) sex determination ranges from homomorphic to heteromorphic female (ZZ/ZW) or male heterogametic (XX/XY) systems, plus potentially polygenic sex determination [40], or even multiple sex chromosomes [41]. Pure temperature-dependent sex determination is relatively rare [42]. Due to advanced genomics approaches the genetic sex determination of all established teleostean model species for ED is known and allows genotypic sexing of undifferentiated or sex-reversed individuals.

The reproductive modes of anurans also cover a wide range of diversity, reaching from aquatic eggs and exotrophic tadpoles to direct development in terrestrial eggs without a larval phase, i.e. lacking tadpoles [43]. Natural parthenogenesis is not known yet in amphibians, but in anurans hybridogenetic systems, including male- or female-biased genetic sex determination, population systems occur [44]. In anurans, auto- and allo-polyploidy are found in several systematic families [45]. By far the most of amphibians feature undifferentiated sex chromosomes and exhibit genetic (genotypic) sex determination, triggered either by male (XY/XX) or female (ZZ/ZW) heterogamety [46], with reports about multiple sex chromosomes [47]. While the majority of amphibians exhibit homomorphic XX/XY or ZZ/ZW sex chromosomes, heteromorphic sex chromosomes have also been reported [48]. The fact that balanced sex ratios are usually observed in clutches suggests that sex determination is genetic in most if not all species. Like in fishes, anurans exhibit a great plasticity for sex differentiation, and thus sex reversal may occur in early developmental stages due to environmental cues. This makes anurans susceptible to ED, affecting reproduction via oestrogenic and androgenic MOA, yet no amphibian species has been reported to exhibit clearly temperature-dependent sex differentiation so far [20]. The best-studied sex determination system in amphibians occurs in the genus *Xenopus*, where coexisting X-, Y- and W-chromosomes are suggested in the diploid species *X. tropicalis* [47], but no master gene has been identified yet. However, in the tetraploid model species *X. laevis, *which exhibits ZZ/ZW-heterogamety, the master gene for sex determination has been characterized as a *Dmrt1*-paralogue, while the W-linked *Dm-w* [47,49] occurs in some closely related species of tetraploid clawed frogs [50,51].

Despite such a great variation and high complexity of genetic sex determination systems in teleosts and anurans, all of these are also characterized by a high degree of plasticity concerning sex differentiation during early developmental phases, where sex reversal or malformation of gonadal and/or potentially phenotypic sex might occur in response to endocrine changes in the environment. Therefore, the susceptibility of both teleosts and anurans to exogenously administered sex steroids, causing sex reversal or developmental problems during the sensitive window for sexual differentiation, means that they can serve as sentinel species for ED detection by exhibiting oestrogenic and androgenic modalities.

In general, the endocrine systems of teleosts and anurans appear relatively comparable, and their functions are similar to all other vertebrates. The regulation of reproduction is mainly accomplished by the hypothalamic–pituitary–gonadal axis, triggering sexual differentiation and regulation of reproduction in amphibians [52,53] and fish [54], comprising targets for ED.

The most superior endocrine centre, the hypothalamus, releases gonadotropin-releasing hormone to the pituitary to trigger the secretion of gonadotropins, luteinizing hormone (LH) and follicle-stimulating hormone (FSH). The gonadotropins stimulate the gonads to synthesize and secrete the sex steroids, androgens and oestrogens, acting on target cells and in turn cause negative feedback on the hypothalamus and pituitary to regulate homeostasis. In anurans, the androgens are the same as those in all higher vertebrates—testosterone and dihydrotestosterone. However, in teleosts, the predominant and most efficient androgen is 11-ketotestosterone, which varies somewhat structurally and might also cause some differences in the efficiency with which EDs act on androgen receptors. Therefore, (anti-)androgenic MOA in anurans might be closer to those of mammals, including humans, than those present in teleosts. In all vertebrates, the only naturally occurring oestrogen is 17ß-estradiol, and oestrogen receptors are thus also remarkably conserved concerning their structures and specificities, suggesting that all vertebrates are susceptible to estrogenic ED. Furthermore, hormone-activating and -inactivating metabolic activities, degradation and excretion processes, as well as binding proteins for transport, also affect the regulation and bioavailability of sex steroids. Therefore sex steroid receptors where ED can mimic a hormone or act as a hormone-antagonist may not be the only targets of ED.

### (b) Impacts of ED on the reproductive biology of teleosts and amphibians

Far more ED research related to oestrogenic and androgenic modalities has been accomplished in teleosts than in anurans, and a higher number of relevant standardized exposure regimes and test guidelines have been addressed for teleosts, including for ecotoxicological model species such as *D. rerio*, *P. promelas*, *O. latipes,* and more recently *G. aculeatus*. These test guidelines or exposure regimes, as mentioned above, range from short- to long-term treatments, the full life cycle or even multigenerational studies. EDs affecting estrogenic MOA have been investigated extensively in fish, where they cause numerous feminization phenomena, depending on life stage [55], affect estrogenic biomarkers such as vitellogenin [56] and also impact their behaviour [57–60]. Estrogen-induced vitellogenin production can be counteracted by anti-estrogens, such that teleosts exhibit susceptibility to both oestrogenic and antioestrogenic MOA [61]. Some EDs can interfere with aromatase, leading to altered oestrogen availability and adverse impacts on teleost reproduction [62]. Androgenic MOA have been clearly demonstrated impairing reproduction, particularly in male teleosts [63,64]. More recently, the three-spined stickleback has been introduced as an excellent model to assess not only (anti)estrogenic but in particular also (anti)androgenic MOA because of its unique purely androgenic biomarker, the androgen-dependent spiggin protein [65,66].

Although the knowledge about EDs affecting the reproductive biology of anurans is much more limited compared to that in teleosts, several reviews describe anurans as good sentinels to assess potential risks for estrogenic and androgenic MOA [20,22,52,67]. The main model species, *X. laevis*, has been well established for assessing reproductive risks in ED, using larval as well as adult exposure regimes that are easy to perform due to the fully aquatic life style of that species [20,68–70]. Estrogenic and androgenic MOA of ED in teleosts and anurans are similar, adversely affecting sexual differentiation, feedback mechanisms on the hypothalamus–pituitary–gonad axis, sex steroid levels, biomarkers such as vitellogenin, and gonadal histomorphology (reviewed by [22]). The potential basic mechanisms underlying sexual differentiation and potential impacts of ED are reviewed in depth by [53], including the ontogenetic courses of the sex steroid-synthesizing enzymes (5α-reductases and aromatase), sex steroid receptors and gonadotropins [71].

More recently, the behaviour of adult *X. laevis,* specifically male advertisement (mating) calls [72–77] as well as female choice experiments [75], revealed that behaviour might have the potential to become the most sensitive biomarker to assess estrogenic and androgenic modalities, even by discriminating between (anti)estrogenic and (anti)androgenic MOA.

In addition, comparative synchronized tadpole exposure experiments have been performed involving the model species *X. laevis* in parallel with non-model anuran species (*Hyla arborea, Bufo(tes) viridis*) that also differed in their genotypic sex determination systems (ZW in *X. laevis*; XY in *H. arborea* and *B. viridis*). This novel approach revealed quantitative differences in genetic-male-to-phenotypic-female sex reversal in non-model versus model species and qualified molecular sexing in ED-analyses as a requirement to identify sex reversals to detect species-specific vulnerabilities to ED in amphibians. These three species showed similar impacts on sexual differentiation among anurans in principle, but different vulnerabilities (e.g. due to deep phylogenetic divergence, differences in life style and history) [78–81], regardless of the great variation in genetic sex determination. Given the great species diversity and the phylogenetic divergence of many of the anuran species (which number over 7500), it can be expected that several of these may be differently susceptible to ED. Thus, data obtained from earlier studies in *X. laevis* in general and without genetic sex information specifically should not be uncritically extrapolated to other systematic anuran families [78].

### (c) The thyroid system of teleosts and anurans

Thyroid systems are restricted in the animal kingdom to all vertebrates and characterized by the presence of a common histological structure, the thyroid follicles, exhibiting a great variety of organizational levels, from dispersed to condensed morphological distributions and even forming distinct thyroid glands. The regulation of thyroid hormones (TH), tetraiodothyronine (T_4_) and triiodothyronine (T_3_), which are associated with metabolism and permissive for various differentiation and developmental processes, is triggered via feedback mechanisms by the hypothalamus–pituitary–thyroid axis, as also described in fishes [82,83] and amphibians [84–86].

The hypothalamus receives sensory inputs from the whole nervous system and in turn secretes releasing factors, acting on hypophyseal thyrotropes to release thyroid stimulating hormone (TSH) into the blood stream to stimulate thyroidal activities. In so-called ‘higher’ vertebrates, TSH-releasing hormone (TRH) is the main stimulator for TSH secretion by the pituitary. However, in teleosts and at least larval amphibians TRH seems to be ineffective or even to be inhibitory. Instead corticotropin releasing hormone is often found to be the main stimulator for TSH release, but it seems that no generalized hierarchic regulation can be stated for TSH, either in teleosts nor in anurans. TSH in turn stimulates TH synthesis through the growth and development of thyroid follicles, which show a great variety of morphological distributions in teleosts but form a discrete thyroid gland in all anurans. Local bioavailability of TH in vertebrates, including teleosts and anurans, is triggered very conservatively by a complex interplay of TH synthesis. This is followed by binding of TH to transporter proteins and specific TH receptors, TH metabolism by deiodinases and excretion of TH. Therefore, the vertebrate thyroid system offers many more targets for ED, finally affecting the bioavailability of TH rather than acting simply through direct interference with TH receptors.

Amphibian metamorphosis is the classical, unique and even drastic example of the endocrine regulation of development by the thyroid system. In anurans, metamorphosis is a general feature triggered by the thyroid system, whereas in teleosts comparable drastic developmental changes requiring TH are less obvious but include metamorphosis in flat fish, obligatory smoltification in anadromic fish (such as salmon), adaptation processes for changing osmoregulation, and colour changes for reproduction. Therefore, in ecotoxicological model species such as *D. rerio*, *P. promelas*, *O. latipes* and *G. aculeatus*, the understanding of functions of the TH system is not yet complete but many efforts have been made to introduce and detect new ecotoxicological endpoints for thyroid disruption besides the evaluation of the thyroid's histopathology.

In the framework of the EU-project ERGO (EndocRine Guideline Optimisation), one main task is to validate and integrate new endpoints into existing OECD test guidelines (TGs) to enable us also to assess teleostean models thyroidal modalities comparatively [87]. The most discussed thyroid-sensitive endpoints include TH, thyroid-related gene expression, thyroid histopathology, eye histopathology and pigmentation, as well as swim bladder inflation [88]. Great emphasis is made on addressing thyroidal impacts on eye histopathology [89,90] and swim bladder inflation [91–93] at very early embryonic life stages, which are not considered to be real animal experimentation. However, in comparison to anuran models, where the specific effects of the thyroid system on metamorphosis are very well understood, it is less clear how specifically and sensitively the suggested new endpoints might be affected by thyroidal modalities in teleosts [88].

### (d) Impacts of ED on the thyroid system of teleosts and anurans

In teleostean model species, the impacts of (anti)thyroidal MOA are less clear compared to anuran metamorphosis being the most drastic short-term organ remodelling in vertebrate development. However, in principle, thyroidal modalities can be determined in teleosts by comparable endpoints, such as TH levels, TH-related gene expression, thyroid and eye histopathology, and eye pigmentation, or by another specific endpoint such as swim bladder inflation, but there is still a need to evaluate the specificity and sensitivity of these biomarkers [88] in comparison to anurans. Looking critically at more recent reports [89,90], it appears rather questionable whether the sensitivity and assessment methods are sufficient to establish teleosts as sound surrogates for anurans for thyroidal ED-testing. In particular, this is because the most specific and reliable endpoints for thyroidal ED are based on the histopathology of the thyroid [94,95] and eye [90], which are very labour-intensive to access and less easy to measure, compared to thyroid histology in anurans, which possess a discrete thyroid gland and not just dispersed thyroid follicles, as in teleosts. In addition, developmental stages of larval anurans can be easily determined by gross morphological landmarks. Environmental pollution by perchlorate has been shown to act as an antithyroidal ED on thyroid follicles in the teleost *Campostoma anomalum*, as well as in the anuran Northern cricket frog, *Acris crepitans* [96], suggesting that this has a reliable transferability from model species to wildlife, at least for thyroid histopathology. Further endpoints that are affected by antithyroidal ED, such as a decrease in the development of the scales and their coloration in *P. promelas* [97], are even harder to quantify in a dose-dependent manner. In addition, inhibition of TH synthesis by perchlorate-induced hermaphroditism impaired reproductive behaviour and swimming performance in the three-spined stickleback [98,99], which indicates strong cross-talk of thyroidal modalities to further endocrine axes or even to the nervous system, raising again some concern about the specificity and sensitivity of (anti)thyroidal endpoints in teleosts.

In amphibians, environmental pollutants acting as EDs [67] have been reviewed and the knowledge about (anti)thyroidal MOA is rapidly increasing because the anuran *X. laevis* is well established as a very sensitive and reliable model organism for the assessment of thyroid system disruption using several OECD TGs. Endpoints and biomarkers include different methodological levels such as gross morphology for staging and thyroid histopathology, but in addition, TH-dependent gene expression and further so-called NAMs (new approach methods) are ongoing to facilitate the detection of various MOA and improve the sensitivity of ED determinations.

### (e) Existing tests and test guidelines for EATS

For human health and environmental risk assessment, EATS [24] are covered by several validated mammalian OECD TGs (all TGs accessible at [100]) assessing estrogenic (output data from ToxCast; ER Bioactivity Model; OECD TG 440: Uterotrophic bioassay in rodents), androgenic (OECD TG 442: Hershberger bioassay), thyroidal (no specific TG yet available *in vitro* or *in vivo* but relevant thyroid parameters determined by OECD TGs 407, 408, 409, 416 or 443, and 451–3), and steroidogenic (OECD TG 456: Steroidogenesis assay; US Environmental Protection Agency (EPA) OPPTS 890.1200: Aromatase assay) modalities.

For non-mammalian vertebrates it is expected by OECD [24] to investigate EATS using aquatic vertebrates, namely teleosts and amphibians, as models. The determination of EAS modalities might be preferably achieved by OECD TG 229 (Fish short term reproduction assay), 230 (21-day fish assay) or 234 (Fish sexual development test). In addition, for assessment of T-modality the OECD TG 231 (amphibian metamorphosis assay (AMA)) should be conducted.

Further OECD TGs using teleosts and amphibians have more recently been developed to completely cover amphibians using African clawed frogs (*Xenopus laevis*) as model organism and EATS by OECD TG 241 (The larval amphibian growth and development assay (LAGDA)) or at least in part thyroidal modalities by OECD TG 248 (*Xenopus* Eleutheroembryonic Thyroid Assay (XETA)). Furthermore, teleosts have been introduced as models covering estrogenic MOA by OECD TG 250 (Detection of Endocrine Active Substances, acting through estrogen receptors, using transgenic tg(cyp19a1b: GFP) Zebrafish embrYos (EASZY)), androgenic MOA by OECD TG 251 (Rapid Androgen Disruption Activity Reporter (RADAR)), and estrogenic and androgenic MOA by OECD TG 240 (Medaka Extended One Generation Reproduction Test (MEOGRT)).

The amphibian OECD TGs 231, 241 and 248 can be used for testing (anti)thyroidal ED, however, their practicability covers a wide range, best described by the varying exposure times of 3 d, 21 d and 112 days for XETA, AMA and LAGDA, respectively. So far, endpoints and biomarkers include only classical methodological levels, such as growth, gross morphology for staging and thyroid histopathology. However, the so-called NAMs—such as genomics, transcriptomics, proteomics and metabolomics—provide a sound potential for establishing more sensitive and reliable diagnostic parameters and should become incorporated in ED TGs wherever possible in order to facilitate the plausibility for detecting specific MOA and to support reduction of test animals. The XETA [101,102], using genetically modified *X. laevis*, has some restrictions, mainly due to the fact that early stages are exposed and still lacking TH biosynthesis, and thus EDs inhibiting TH formation are not accessible. Therefore, AMA [103–105] and LAGDA [106] provide assessments of all (anti)thyroidal MOA with high sensitivity, if all suggested endpoints in AMA are determined in parallel. However, AMA has a much shorter exposure time compared to LAGDA, which might provide an additional merit to potentially also address sexual differentiation. A more recent attempt [107] aims to improve AMA as extended AMA (EAMA). However, it seems that the best compromise for a TG to assess both (anti)thyroidal modalities and sexual differentiation sensitively, as well as making a first evaluation of gametogenesis, could be accomplished by shortening the exposure regime for SEXDAMAX (sexual development and metamorphosis assay *Xenopus*) [108,109] from 75 to 35 days but to start chemical exposure later at NF stages 51/52, according to AMA (TG 231).

## 4. Non-EATS modalities

EATS modalities [29,30] cover only a part of the endocrine systems associated with reproduction via sex steroids, steroidogenesis and metabolism, as well as development (e.g. metamorphosis) being governed by the thyroid system. However, further endocrine pathways are present and resemble potential targets for ED. The potential for non-EATS modalities to also become a subject for ED research was mentioned quite early on [20,25], and has recently been reviewed [26], there including endocrine pathways such as retinoic acid, peroxisome proliferator-activated receptors (PPARs), insulin receptor signalling, gastrointestinal hormones and cardiovascular-related hormones. It is suggested that even further endocrine systems are impacted by as-yet only partly identified ED [20,22,25] that work on stress axes regulating catecholamines or gluco- and mineralo-corticosteroids, (anti)gestagenic MOA and growth axis, acting via growth hormone and insulin-like growth factors. Recently, special emphasis has been put on the so-called metabolic syndrome, affecting mainly the liver, which is a good endocrinological example of an organ that is affected by multifactorial drivers and resulting in similar endpoints of adverse outcome pathways, for instance steatosis. In anurans, larval exposure to bisphenol A induced hepatic gene expression of IGF-1, revealing also somatic growth [110], and EDs have been reported to induce unexpected metabolic disorders [111] or to disrupt multiple endocrine axes [112]. Gestagenic MOA are one of the most neglected MOA, not covered by EATS. One such example is progestins, such as Levonorgestrel, which act via gestagenic MOA and are commonly used for contraception in the **‘**mini-pill’. Even at very low concentrations, these substances have been reported to have especially drastic effects on reproduction in teleosts [113]. By contrast, anurans responded less sensitively concerning their sexual differentiation [114,115] but the disruption of their thyroid system [116] and mating call behaviour [74] was surprisingly more pronounced, and the latter finding might be explained by the partly androgenic activity of Levonorgestrel. This topic has extensively been reviewed [117], providing evidence that progestins adversely affect teleosts and anurans by multiple MOA, including thyroidal disruption. Although it is obvious that Levonorgestrel has drastic effects on the thyroid system in anurans, no further research has been undertaken to clarify that for mammals, including humans.

In general, the complexity and high probability that various endocrine systems interfere strongly by cross-talking to each other, as well as interacting with the nervous and the immune systems, makes it difficult to disentangle single specific endocrine disrupting pathways by developing validated TGs for the assessments of specific MOA of ED. Furthermore, a single substance or its metabolites might also interfere with various endocrine systems or MOA, as shown by different testing methods.

## 5. Environmental factors causing endocrine disruption

The majority of ED research is dedicated to investigate direct adverse endocrine impacts on wildlife and humans of synthetic compounds of anthropogenic origin. However, there is evidence that as well as man-made chemicals, natural substances also exist that interfere as EDs with the endocrine systems. Such natural compounds are numerous, occur in great variety, and—as in humans—they are supposed to become incorporated into animals mainly via water, diets and/or cosmetics, e.g. phytoestrogens such as genistein, daidzein [118] and resveratrol [119]. The latter one even exhibits disrupting effects on various endocrine systems. Despite the ubiquitous presence of natural, mainly plant-based compounds exhibiting potential to act as ED, their concentrations might only reach effective concentrations for ED if they are provided in a modified way due to anthropogenic activities. Therefore, artificially phytoestrogen-enriched cosmetics have to keep thresholds to be safe for humans [118], but the use of soya bean-based diets might bear a risk for reproduction in ruminants if applied too intensely by farmers [120]. In teleosts and anurans, a shift of diets from animal protein towards soya bean-based diets might also bear a risk for reproduction, especially in aquaculture, due to estrogenic MOA. Nevertheless, although such natural substances have comparable endocrine effects in teleosts [121] and anurans, where—in addition—thyroid system disruptive impacts have been found (reviewed in [122]), under conditions in the wild, it is questionable whether any exposure scenario might reach endocrine-effective concentrations.

Therefore, to elucidate whether might become exposed to natural ED at relevant concentrations, aqueous extracts from ground leaves of reed, beech and oak, collected in nearly undisturbed habitats, were assessed concerning their ED potential [123]. These aqueous extracts displayed low estrogenic but strong antiandrogenic MOA, shown by yeast estrogen (YES) and androgen (YAS) screening assays *in vitro* that depended in strength on the plant species. The most potent oak leaf extract has also been assessed concerning sexual differentiation by exposing larval *X. laevis* to concentrations of dissolved organic carbon ranging from 0.1 to 50 mg l^−1^, i.e. within naturally occurring levels. In male tadpoles, the leaf extract revealed disruptive development of testes by formation of additional lacunae starting at 10 mg l^−1^, and even some sex reversal occurred, as indicated by testicular oogonia at the highest concentration. To counteract the antiandrogenic MOA in male tadpoles, hypophyseal LH gene expression at 50 mg l^−1^ has been elevated. Thus, such a natural exposure scenario—which might occur regularly in small ponds within oak forests—can affect not only anuran development but also that of fishes via moderate estrogenic and strong antiandrogenic MOA leading to feminizing effects. Keeping in mind a scenario where an environmental factor can lead to proper ED-responses, further efforts should be made to understand how environmental factors might interfere with ED systems by extending research to other vertebrates and invertebrates as well. However, due to the fact that such natural exposures last as co-evolutionary traits, such ED risks might affect only locally biodiversity but might not pose any general future threat.

The well-described scenarios for climate change suggest temperature as an environmental factor that will lead to endocrine responses. In teleosts, temperature-dependent sex determination is rare and restricted to only a few species [42,124], whereas in amphibians no temperature-dependency of sexual differentiation has been reported. However, it is well-known that temperature can override the primary genetical sex signals in amphibians and thereby cause a sex-bias (reviewed by [125,126]). This may occasionally and naturally occur and lead to sex-reversal, here understood as a contradiction between genotypic and phenotypic sex [127,128]. Therefore, temperature has to be considered as an environmental factor for endocrine responses and controlled laboratory experiments are needed to elucidate its impacts. Beyond potential impacts from climate change with the rising of average and especially extreme temperatures, teleosts and amphibians may suffer even more in the future from changes in salinization, for example, and destruction of habitats or fostering the occurrence of diseases.

Next to bacterial and viral diseases, which might be fostered directly or indirectly by environmental factors such as temperature, parasites are another important natural source to impact teleosts and amphibians even via endocrine responses. Among fish parasites, the tapeworm *Ligula intestinalis* and microsporidae have been suspected to act on endocrine systems in teleosts, adversely affecting their reproductive physiology [129,130]. *L. intestinalis* can exhibit high prevalence in its hosts, chub (*Squalius cephalus*) and roach (*Rutilus rutilus*), suggesting that it might become relevant at the population level due to a hampered reproduction. Field and laboratory studies revealed that ligulosis can even completely block gonadal development by reduction of gonadotropin gene expression and accordingly reduce sex steroid levels in male and female hosts [131–135].

In amphibians, the induction of endocrine stress has been linked to environmental pollution, which in turn might affect the immune system and result in a higher susceptibility for infectious diseases like chytridiomycosis [136] and parasites, i.e. nematodes, cestodes and trematodes [137–140]. However, *vice versa* parasitic infection by the trematode *Ribeoroia ondatrae* can act as a natural ED, causing morphological deformities in amphibians that look similar to ED-phenomena and are induced via disruption of retinoic pathways [141,142]. Therefore, for ED assessment in wildlife, it should be emphasized that only non-parasitized individuals should be used for determining biomarkers related to ED.

Environmental factors to control fish reproduction include as main natural triggers the temperature and light regime in a species-specific manner and also food availability is important, but environmental pollution is an artificial man-made factor [143]. The fact that the light regime affects the endocrine responses associated with reproduction, especially in seasonally spawning fish, has been used for aquacultural manipulation of fish maturity [144,145]. It has already been proposed that artificial light at night (ALAN) might bear the risk of interfering with endocrine systems in several organismic groups [146], and thus exhibit the potential to act as ED, especially in fishes. We emphasize that light pollution via ALAN is not a pathway involving the exposure to an **‘**exogenous substance’ [10] but rather to the physical force of electromagnetic radiation. Therefore, the recent WHO definition does not cover ALAN as an environmental factor to act as ED, implying a need to evaluate whether ALAN bears a risk for disrupting endocrine systems in fish and amphibians. To assess ALAN at realistic exposure scenarios with low levels of illumination, its impact on native European fish species such as roach (*R. rutilus*) and perch (*Perca fluviatilis*) has been evaluated, to understand endocrine impacts on epiphyseal melatonin secretion, on levels of the stress hormone cortisol and the potential of indirect impacts on gonadotropin gene expression, sex steroids and TH [147–154]. Melatonin secretion under laboratory conditions has already been shown to be significantly affected at the lowest applied ALAN of 0.01 lux [152], whereas even higher exposures to ALAN did not elicit any stress response via cortisol [148]. In addition, reductions in gonadotropin gene expression levels, as well as of sex steroid levels, have been assessed in both laboratory [149,150] and real-world experiments [151]. Disruption of the TH balance seems also to be an adverse outcome by ALAN [153] and, furthermore, impacts on the immune system and oxidative stress have been suggested [154]. In summary, even under realistic exposure scenarios, ALAN is a strong ED, affecting melatonin levels and thereby potentially indirectly disrupting the reproductive physiology in fish. Compared to fishes, in amphibians, ALAN research data are barely available, and an assessment of MOA is missing [155] but it can be assumed that ALAN might also interfere in a comparable way to fish.

## 6. General discussion and conclusion

The emerging global issue of environmental pollution presents one human impact for which no comprehensively characterized planetary boundaries have been accepted [6,7] and one important subtopic since the 1990s comprises endocrine disruption. Its adverse outcome pathways affect wildlife and humans indirectly via disruptions of endocrine systems rather than via direct toxic impacts that could ease risk assessments. However, despite plenty of efforts to determine in a validated framework specific MOA, associated with endocrine disruption for regulatory purposes, such research directions are facing several problems. The complexity of endocrine interconnections exhibits a crosstalk between several endocrine axes or systems that cannot be separated from each other. Therefore, only some substances or one environmental factor, e.g. temperature or ALAN, may affect very specifically a single endocrine system by one MOA. In addition, it should be considered that a single ED has the potential to interfere with several endocrine systems in parallel or to interact directly or indirectly with their crosstalk. In turn, this complexity hampers the idea of assessing ED compounds and factors of concern as acting as a specific effector for endocrine systems through a single characteristic MOA. The trial of using EATS modalities for risk assessment is a starting point, but even this approach faces the problem that several substances can address and affect several endocrine systems simultaneously.

The efforts to also include non-EATS modalities for the establishment of specific TGs are a logic prerequisite to addressing the whole issue of ED. However, from an endocrinological point of view, such a complexity of crosstalk—which even exhibits impacts across various endocrine systems in parallel, as well as with the immune and nervous systems—requires test approaches to evaluate crosstalk-networks at once. In accordance with the 3R-strategy (reduce, refine, reset), the recent mindset supports approaches that determine all toxicological and ecotoxicological effects in testing batteries *in vitro* rather than using *in vivo* models for animal experimentation. However, it has to be considered that so far only whole animal models *in vivo* can really tell us what might happen to an ‘intact’ organism at several organizational levels, as discussed above. The amazing progress in being able to introduce organoid cultures reconstituting an endocrine organ for instance thyroid [156,157], or even multi-organoids-on-a-chip [158] using mammalian stem cells—offers fascinating opportunities for combining even various (endocrine) organ systems within one *in vitro* system. The development of such organoids-on-a-chip might boost our principal understanding of how several endocrine systems can crosstalk with each other. Nevertheless, the classical endocrine systems are regulated via feedback mechanisms including the levels of hypothalamic and pituitary hormones, which might be the greatest challenge in reconstituting a classical endocrine system completely.

The existing OECD strategy to assess risk for EATS modalities [24] is fostering continuing improvement for ED assessment, but it might be warranted to get regular periodic updates because the current knowledge is growing rapidly. Especially for non-target organisms, like teleosts and amphibians, the existing TGs often reveal results as being ‘sensitive to, but not diagnostic of EATS' modalities. For instance, the more recent attempts to characterize (anti-)gestagenic MOA on reproductive parameters are not covered by specific *in vitro* TGs, and the drastic effects of the progestin levonorgestrel on the reproductive and thyroid systems could only be determined through *in vivo* exposures of anurans. For formalistic reasons, ED impacts via gestagenic MOA should be assigned to the aforementioned non-EATS modalities. However, it might be better to integrate (anti-)gestagenic MOA as an extension for existing EATS-related TGs, covering impacts on both reproduction and the thyroid system. Non-EATS modalities in teleosts and amphibians are more numerous than EATS and show often a more complex adverse outcome pathway network. This can be impacted at various levels by multiple exogenous or endogenously interacting factors, e.g. the metabolic syndrome. Therefore, instead of compiling steadily increasing numbers of linear AOPs in AOP Wiki, which very often do not specifically distinguish between ED and general toxicity, we suggest that work should continue on AOP networks, unraveling ED effects that are associated with the same outcome by using various MOA. The continuous integration of new findings in such networks might be more helpful for regulators, providing them with a sound overview about AOPs related to one endocrine system instead of having an increasingly growing catalogue of various AOPs that are only potentially associated with one system.

Teleosts and amphibians, serve as sensitive sentinels for ED in wildlife as well as model species for risk assessment in TGs. Recent approaches aim to beneficially optimize existing TGs—for instance to integrate thyroidal ED assessment into the OECD TG 236 (FET). Impacts on swimbladder inflation and eye development have been shown, but it seems questionable whether a similar sensitivity and specificity to those found in amphibians could be accomplished. Thus, there is an urgent need to compare teleost and amphibian TGs to establish whether amphibian testing could be reduced in favour of an extended version of FET, or *vice versa* to extend the AMA being proposed as EAMA [107] to reach developmental stage 62 or alternatively to expose until completion of metamorphosis (stage 66). Such extended AMAs would enable not only the parallel assessment of thyroidal disruption and sexual differentiation, but might also provide sufficient information to abolish or reduce the need for fish testing to assess reproduction. The high sensitivity of gonadal plasticity in adult *X. laevis* [69,70] might even be a reason to go further to establish a new 21 d TG to assess impacts on the reproductive physiology of adult frogs. The behavioural male mating call test [159], detecting MOA non-invasively within a few days of exposure, might be an attractive option if artificial intelligence-based algorithms become available to discriminate and evaluate the different calling types for each individual in an automated way.

From an endocrinological point of view, assessment of endocrine systems including feedback mechanisms still requires whole animal exposures. The established methods to determine genetic sex or so-called NAMs might open an option to drastically reduce the numbers of exposed individuals, especially addressing sexual differentiation and sex reversal, and thus the design of AMAs should be reconsidered.

Since ALAN can act as environmental factor, i.e. as an ED affecting primarily melatonin levels and indirectly affecting reproduction success in teleosts, such as perch and roach, this demonstrates that not only substances but also a further physical force—electromagnetic radiation as visible light—can act as an ED. Further examples of EDs that are caused by parasitic infections also indicate that it is not only substances that can act as EDs. Therefore, we suggest an adaptation of the generally accepted WHO-definition for ED [10] by replacing the term ‘exogenous substance’ with ‘environmental factors, e.g. exogenous substances of natural and anthropogenic origin, parasitic diseases and ALAN’. The naturally occurring EDs, such as organism-based compounds or parasitic infections, have been involved in coevolutionary processes for ages, and thus without resulting in a general loss of biodiversity. Therefore, to address the ‘One Health’-principle, we advocate the minimization of general anthropogenically induced factors, especially chemical pollution by synthetic or even natural substances, and now to change our focus to ALAN to avoid and fight endocrine disruption.

## Data Availability

This article has no additional data.
